# Functional Diversity of Evolutionary Novelties: Insights from Waterfall-Climbing Kinematics and Performance of Juvenile Gobiid Fishes

**DOI:** 10.1093/iob/obz029

**Published:** 2019-11-21

**Authors:** R W Blob, R Lagarde, K M Diamond, R M Keeffe, R S Bertram, D Ponton, H L Schoenfuss

**Affiliations:** 1 Department of Biological Sciences, Clemson University, Clemson, SC 29634, USA; 2 Hydrô Réunion, Z.I. Les Sables, 97427 Etang Salé, La Réunion, France; 3 Université de Perpignan Via Domitia - CNRS, Centre de Formation et de Recherche sur les Environnements Méditerranéens, UMR 5110, F 66860 Perpignan, France; 4 Department of Biology, University of Florida, Gainesville, FL 32611, USA; 5 Aquatic Toxicology Laboratory, St. Cloud State University, St. Cloud, MN 29634, USA; 6 ENTROPIE, IRD, Université de La Réunion, CNRS, Laboratoire d’Excellence CORAIL, c/o Institut Halieutique et des Sciences Marines (IH.SM), Université de Toliara, Route du port, Toliara, P 141, 601 B, Madagascar

## Abstract

The evolution of novel functional traits can contribute substantially to the diversification of lineages. Older functional traits might show greater variation than more recently evolved novelties, due to the accrual of evolutionary changes through time. However, functional complexity and many-to-one mapping of structure to function could complicate such expectations. In this context, we compared kinematics and performance across juveniles from multiple species for two styles of waterfall-climbing that are novel to gobiid fishes: ancestral “powerburst” climbing, and more recently evolved “inching”, which has been confirmed only among species of a single genus that is nested within the clade of powerburst climbers. Similar net climbing speeds across inching species seem, at first, to indicate that this more recently evolved mode of climbing exhibits less functional diversity. However, these similar net speeds arise through different pathways: *Sicyopterus stimpsoni* from Hawai’i move more slowly than *S. lagocephalus* from La Réunion, but may also spend more time moving. The production of similar performance between multiple functional pathways reflects a situation that resembles the phenomenon of many-to-one mapping of structure to function. Such similarity has the potential to mask appropriate interpretations of relative functional diversity between lineages, unless the mechanisms underlying performance are explored. More specifically, similarity in net performance between “powerburst” and “inching” styles indicates that selection on climbing performance was likely a limited factor in promoting the evolution of inching as a new mode of climbing. In this context, other processes (e.g., exaptation) might be implicated in the origin of this functional novelty.

## Synopsis

**FRENCH:**

Diversité fonctionnelle des innovations évolutives: l’exemple de la cinématique et des performances de grimpe des chutes d’eau des juvéniles de gobies **Résumé** L’évolution de nouveaux traits fonctionnels peut contribuer significativement à la diversification des lignées. Les traits fonctionnels les plus anciens peuvent montrer plus de variabilité que les plus récents du fait de l’accumulation de changements évolutif au cours du temps. Cependant, ces prédictions peuvent être complexifiées par la diversité des fonctions et par l’implication de plusieurs structures dans une même fonction. Dans ce contexte, nous avons étudié la cinématique et les performances de grimpe des chutes d’eau de plusieurs espèces de gobies utilisant deux styles de grimpe originaux au sein de cette famille: le mode « powerburst » plus ancestral et le mode « inching » qui a évolué plus récemment. Le mode inching n’a été confirmé que pour deux espèces du même genre incluses au sein du clade des powerburst. Des vitesses de grimpe similaires entre les espèces utilisant le mode inching paraissent indiquer que ce mode de grimpe, qui a évolué plus récemment, présente une diversité fonctionnelle moins élevée. Toutefois, la similarité des vitesses de grimpe entre les deux espèces s’explique par des processus différents: le *Sicyopterus stimpsoni* d’Haiwaï se déplace plus lentement que le *S. lagocephalus* de La Réunion mais passe plus de temps en mouvement. La production de performances similaires, résultant de processus différents, reflète un phénomène semblable à celui de l’implication de plusieurs structures dans une même fonction. Si les mécanismes sous-jacents ne sont pas explorés, ces similarités peuvent perturber l’interprétation des différences relatives de diversité fonctionnelle entre les lignées. Par ailleurs, les performances de grimpe similaires entre certaines espèces utilisant le mode inching et d’autres le mode powerburst paraissent indiquer que la force de sélection sur les performances de grimpe est sans doute un facteur réduisant l’avantage évolutif du mode de grimpe inching. Dans ce contexte, d’autres mécanismes (e.g., exaptation) pourraient être à l’origine de cette innovation fonctionnelle. Translated to French by Raphael Lagarde (raph.lagarde@gmail.com)

**GERMAN:**

Die Funktionelle Leistung von Evolutionären Neuheiten: Erkenntnisse durch Kinematische- und Leistungsstudien von Wasserfallklettern an jugendlichen Gobiid-Fischen **Zusammenfassung** Die Entwicklung neuartiger funktioneller Merkmale kann wesentlich zur Diversifizierung von Abstammungslinien beitragen. Ältere funktionelle Merkmale können aufgrund der im Laufe der Zeit auftretenden evolutionären Veränderungen größere Unterschiede aufweisen als neuere Merkmale. Die funktionale Komplexität und die Eins-zu-Eins-Zuordnung von Struktur-zu-Funktion können solche Erwartungen jedoch erschweren. In diesem Zusammenhang haben wir die Kinematik und Leistung von Jungtieren verschiedener Spezien von Gobiid-Fischen für zwei Arten des Wasserfallkletterns verglichen: das ältere „Powerburst “-Klettern und das in jüngster Zeit entwickelte „Inching“, das nur in einer Gattung von Gobiid- Fischen vorkommt, die in der Gruppe der Powerburst-Kletterer eingeschlossen ist. Ähnliche Netto-Klettergeschwindigkeiten bei “Inching”-Spezien scheinen zunächst darauf hinzudeuten, dass diese neuere Art des Kletterns wenig funktionelle Vielfalt aufweist. Allerdings ist die ähnliche Nettogeschwindigkeit das Resultat von verschiedenen Faktoren: *Sicyopterus stimpsoni* aus Hawaii bewegt sich langsamer als *S. lagocephalus* aus La Réunion aber bewegt sich mehr kontinuierlich. Das Ergebnis einer ähnlichen Leistung durch zweierlei Funktionspfade ähnelt dem Phänomen der Eins-zu-Eins Zuordnung von Struktur-zu-Funktion. Eine solche Ähnlichkeit kann die angemessene Interpretation der relativen funktionalen Vielfalt zwischen den Linien erschweren, sofern nicht die der Leistung zugrundeliegenden Mechanismen untersucht werden. Insbesondere zeigt die Ähnlichkeit in der Nettoleistung zwischen “Powerburst” - und “Inching” -Kletterstilen, dass die Entwicklung des neuen Kletterstils wahrscheinlich wenig mit verbesserter Kletterleistung zu tun hat. In diesem Zusammenhang könnten andere Prozesse (z. B. Exaptation) an der Entstehung dieser funktionellen Neuheit beteiligt sein. Translated to German by Heiko Schoenfuss (hschoenfuss@stcloudstate.edu)

**PORTUGESE:**

Diversidade funcional de novidades evolucionárias: percepções da cinemática da escalada em cascatas e desempenho de peixes juvenis gobiídeos **Resumo**A evolução de novos traços funcionais pode contribuir substancialmente para a diversificação de linhagens. Os traços funcionais mais antigos podem mostrar maior variação do que as novidades desenvolvidas mais recentemente, devido ao acúmulo de mudanças evolutivas ao longo do tempo. No entanto, a complexidade funcional e os inúmeros mapeamentos de uma estrutura para uma única função podem complicar essas expectativas. Nesse contexto, comparamos a cinemática e a performance em juvenis de várias espécies para dois estilos de escalada em cascata que são novidades em peixes gobiídeos: a ancestral escalada por “explosão” e o evolutivamente mais recente chamado de “avançamento”, que foi confirmado apenas entre espécies de um único gênero que dentro do clado de escaladores por explosão. Velocidades finais de escalada semelhantes entre espécies usando “avançamento” parecem, inicialmente, indicar que esse modo de escalada evolutivamente mais recente exibe menor diversidade funcional. No entanto, essas velocidades finais similares ocorrem por diferentes formas: Sicyopterus stimpsoni do Havaí se move mais devagar que *S. lagocephalus* das Ilhas Reunião, mas pode gastar mais tempo se movendo. Desempenhos semelhantes entre várias vias funcionais refletem uma situação que se assemelha ao fenômeno de mapeamento de uma estrutura para um única função. Essa semelhança tem o potencial de ocultar interpretações apropriadas sobre relativa diversidade funcional entre linhagens, a menos que os mecanismos que afetam o desempenho sejam explorados. Mais especificamente, a semelhança no desempenho final entre os estilos “explosão” e “avançamento”indica que a seleção na performance em escalada provavelmente foi um fator limitante na promoção da evolução por “avançamento” como um novo modo de escalada. Nesse contexto, outros processos (e.g., exaptação) podem estar relacionados com a origem dessa novidade funcional. Translated to Portuguese by Diego Vaz (dbistonvaz@vims.edu)

**JAPANESE:**

進化的ノベルティの機能的多様性：滝登りハゼの稚魚による岩登り時の運動学とパフォーマンスからの洞察 （Abstract: 梗概） 進化的に新しい機能特性は系統の多様化に大きく貢献することが可能である。より古い機能特性は、時間の経過に伴う進化的変化の蓄積により、より新しく発現した特性よりも大きな偏差を示すと考えられるのであるが、機能の複雑さと、構造と機能における多対一の関連性により、この様な予測はより複雑になる可能性がある。これを踏まえて、我々は祖先的岩登りスタイルの“パワーバースト”タイプとその系統群の中から進化的に新しく発現した単一の属が獲得したとされる“インチング”タイプを示す複数種の滝登りハゼの稚魚による岩登り時の運動学とパフォーマンス比較分析した。“インチング”タイプの実質的なクライミング速度はどれも種間の差異が認められなかった、そのためこのもっとも最近進化的に発現した岩登りのスタイルは、機能的多様性をあまり示さないことを示唆しているよう考えられる。しかし、ハワイ島の*Sicyopterus stimpsoni*は、レユニオン島の*S. lagocephalus*よりも遅いが、クライミング移動により多くの時間を費やす傾向にあることが分かった。したがって、この種間に差異のない実質的速度は異なる経路を介して発生していると考えられ、複数の機能経路間で同様のパフォーマンスが得られたことは、多対一の関連性があると言える。そしてこれはパフォーマンスの基礎となるメカニズムが明確にされない限り、系統間の相対的な機能的多様性の適切な解釈を複雑にするであろう。より具体的に言うなれば、“パワーバースト”タイプと“インチング”タイプの実質的パフォーマンスの類似性は、クライミングパフォーマンスのへ淘汰が進化的に新しい岩登りスタイルとしての“インチング”タイプを確立する上で制限要因になり得る可能性が高いことを示している。さらには、他のプロセス（例えば、外適応）がこの機能的ノベルティの起源に関係している可能性があると考えられる。 Translated to Japanese by Takashi Maie (maie.t@lynchburg.edu)

## Introduction

The evolution of novel functional abilities is widely regarded as a key factor that can influence the ecological diversification of lineages (Liem 1973; Wainwright 2007). Through the evolution of novel functional capacities, opportunities to exploit new resources or habitats may open, promoting diversification through either the advent of new specializations, or the radiation of taxa within a lineage (e.g., Konow et al. 2008). Alternatively, some functional novelties may have little impact on diversification (e.g., Price et al. 2010; Wainwright and Price 2016), or even reduce aspects of functional diversity in a lineage (e.g., Higham et al. 2015).

Numerous factors might affect how novel traits contribute to ecological and functional diversification (Vermeij 2001). For example, older traits might show greater functional variation due to the accrual of evolutionary changes through time (Wainwright and Price 2016). Alternatively, novel functional capacities could emerge through evolutionary transitions that pass through a flexible, intermediate range of performance between lower and higher extremes (Blob 2001). In such cases, recently evolved novelties might show greater variation in performance than older traits. Beyond such considerations, the complexity of many functional systems (Wainwright 2007) leads to a potential for multiple structural arrangements of features to produce similar functional performance (many-to-one mapping of structure to function: Alfaro et al. 2005; Wainwright et al. 2005). Such circumstances could further complicate expectations for the functional diversity that would be exhibited by novel evolutionary traits.

**Fig. 1:**
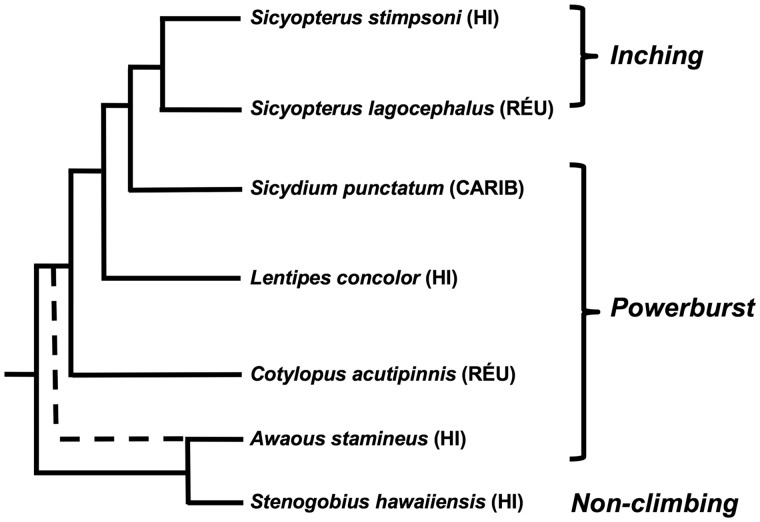
Phylogenetic relationships of gobiid species from which climbing performance has been evaluated, based on previous published analyses (Taillebois et al. 2014). Geographic location of each taxon is indicated in parentheses after its name: HI, Hawai’i; RÉU, La Réunion; CARIB, Caribbean (Dominica). Modes of climbing are indicated to the right of species names. *Stenogobius hawaiiensis* is included for reference as a non-climbing outgroup taxon. Dashed line indicates differing phylogenetic relationships of the genus *Awaous*. Based on available data, inching evolved once within the genus *Sicyopterus*.

The waterfall-climbing abilities found in multiple species of gobiid fishes represent a remarkable functional novelty, through which potential patterns of diversification in functional performance can be tested. Waterfall-climbing is common among amphidromous juvenile gobies that are returning to adult stream habitats. After completing a marine larval phase, climbing is facilitated by a ventral sucker comprised of the fused pelvic fins (Schoenfuss and Blob 2003; Blob et al. 2006; Schoenfuss et al. 2011). Previous observations identified two distinct modes of climbing used by different species of gobies. “Powerburst” climbers use pectoral fin adduction and bursts of axial undulation to propel themselves upwards between periods of attachment to the substrate with the pelvic sucker. In contrast, “inching” climbers sequentially detach and reattach the pelvic sucker and a novel, oral sucker to climb, with little axial movement. Inching has been confirmed only in the genus *Sicyopterus* (Schoenfuss and Blob 2003; Blob et al. 2006; Schoenfuss et al. 2011; Maie et al. 2012), which is phylogenetically nested within several powerburst outgroups (Taillebois et al. 2014; Fig. 1). Thus, powerburst climbing appears to be ancestral, and inching more recently evolved (Cullen et al. 2013).

Comparisons among powerburst species from Hawai’i and the Caribbean have shown a wide range of performance within this climbing mode. However, inching performance has only been measured in one species, *S. stimpsoni* from Hawai’i (Schoenfuss and Blob 2003; Blob et al. 2006; Schoenfuss et al. 2011). To evaluate whether more recently evolved novel traits, like inching, might show less functional diversity than ancestral traits, like powerburst climbing, we compared our previous data on climbing kinematics and velocity from Hawaiian and Caribbean species (Schoenfuss and Blob 2003; Blob et al. 2006; Schoenfuss et al. 2011) to new data from two additional species from the Indian Ocean island of La Réunion: the inching climber *S.lagocephalus*, and the powerburst climber *Cotylopus acutipinnis*.

## Materials and methods

Experiments were conducted under St. Cloud State University Institutional Animal Care and Use Committee guidelines (protocol 12-07). Sampling in La Réunion was conducted under permit N°15-002/DEAL/SEB/UPEMA issued by Direction de l’Environnement de l’Aménagement et du Logement de La Réunion.

Juvenile *S. lagocephalus* and *C. acutipinnis* were collected by electroshocking from the St. Etienne River in La Réunion during April 2015. Fish were collected near the river mouth to ensure that they were recent, postmetamorphic recruits that had not previously climbed. Fish were transported by car to the Hydro Réunion laboratory facility, where they were maintained in aerated stream water at ambient temperature (∼20°C) and a 12:12 h cycle of room lighting. Fish were acclimated a minimum of 24 h prior to testing, which proceeded for 1–2 days after collection. Rocks were placed in enclosures to provide cover and a source of algal growth for food.

**Fig. 2:**
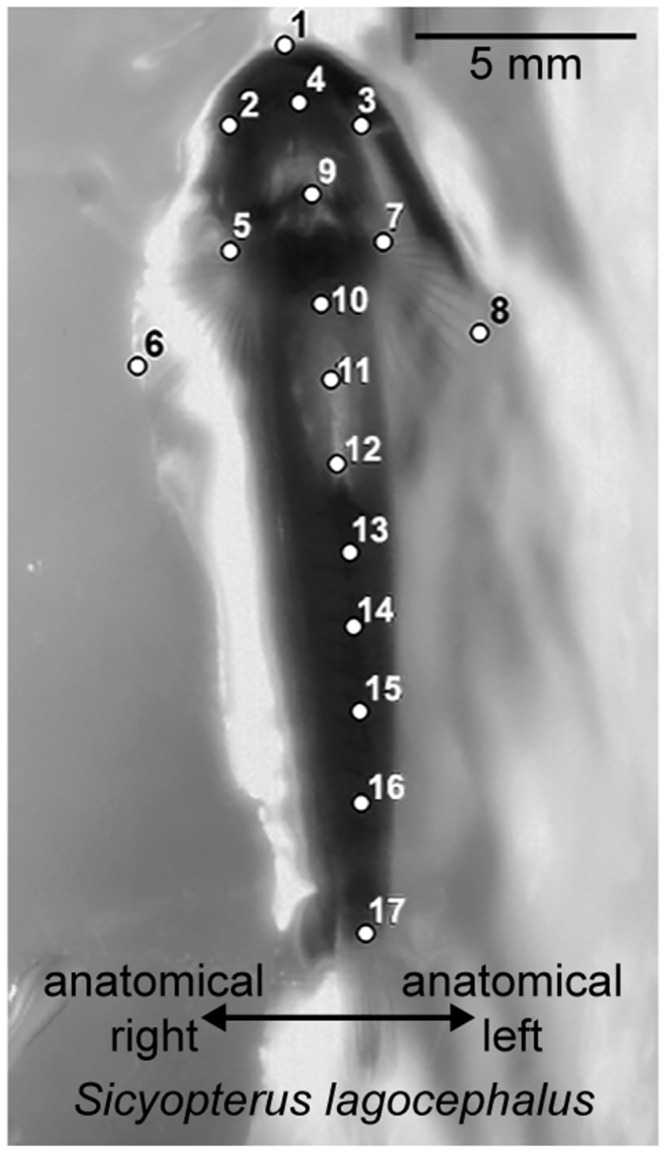
Still image of juvenile *S. lagocephalus*, extracted from high-speed video footage, illustrating the anatomical landmarks that were digitized for kinematic analyses. Because the fish is filmed in ventral view through Plexiglas, references will be made to anatomical left and right, which are opposite of what they appear in the image. (1) anterior midline edge of upper lip; (2) right edge of lip; (3) left edge of lip; (4) posterior midline of oral sucker (for *S. lagocephalus*) or head (for *C. acutipinnis*); (5) base of right pectoral fin; (6) tip of right pectoral fin; (7) base of the left pectoral fin; (8) tip of the left pectoral fin; (9) anterior edge of pelvic sucker; (10–16) evenly spaced body axis points; (17) caudal peduncle.

To facilitate comparisons across species, kinematic and performance data from La Réunion gobies were collected and analyzed following methods of previous studies as closely as possible (Schoenfuss and Blob 2003; Blob et al. 2006; Schoenfuss et al. 2011). Briefly, the kinematics of individual climbing cycles were recorded in ventral view using high-speed video (100–200 Hz, Fastec Highspec 2G) as groups of fish ascended an inclined panel of Plexiglas (52°, lightly roughened with sandpaper) with water flowing as a sheet over its surface (180 mL s^−1^). Lighting was provided by light-emitting diode (LED) fixtures or ambient room lighting. Measurements were performed by tracking landmarks on the head, fins, body axis and suckers (Fig. 2) using DLTdv5 for MATLAB (Hedrick 2008), and using the coordinate data for these landmarks to calculate kinematic variables in MATLAB (Schoenfuss and Blob 2003; Schoenfuss et al. 2011). Performance trials were recorded in dorsal view with Sony Handycam mini-DV cameras (60 Hz), over a standard distance of 20 cm (∼10 body lengths), as fish ascended an inclined (70°), textured plastic chute with water flowing down its surface (Blob et al. 2006). Video records were used to determine the portion of time each fish spent moving versus resting as it ascended the 20 cm distance, from which we calculated climbing speeds while moving and net climbing speeds including rest time. Performance metrics were compared across species via Kruskal–Wallis analyses with Dunn’s post-hoc tests corrected for multiple comparisons, using the Prism 6.1 statistical package (Graph-Pad software).

## Results

**Fig. 3:**
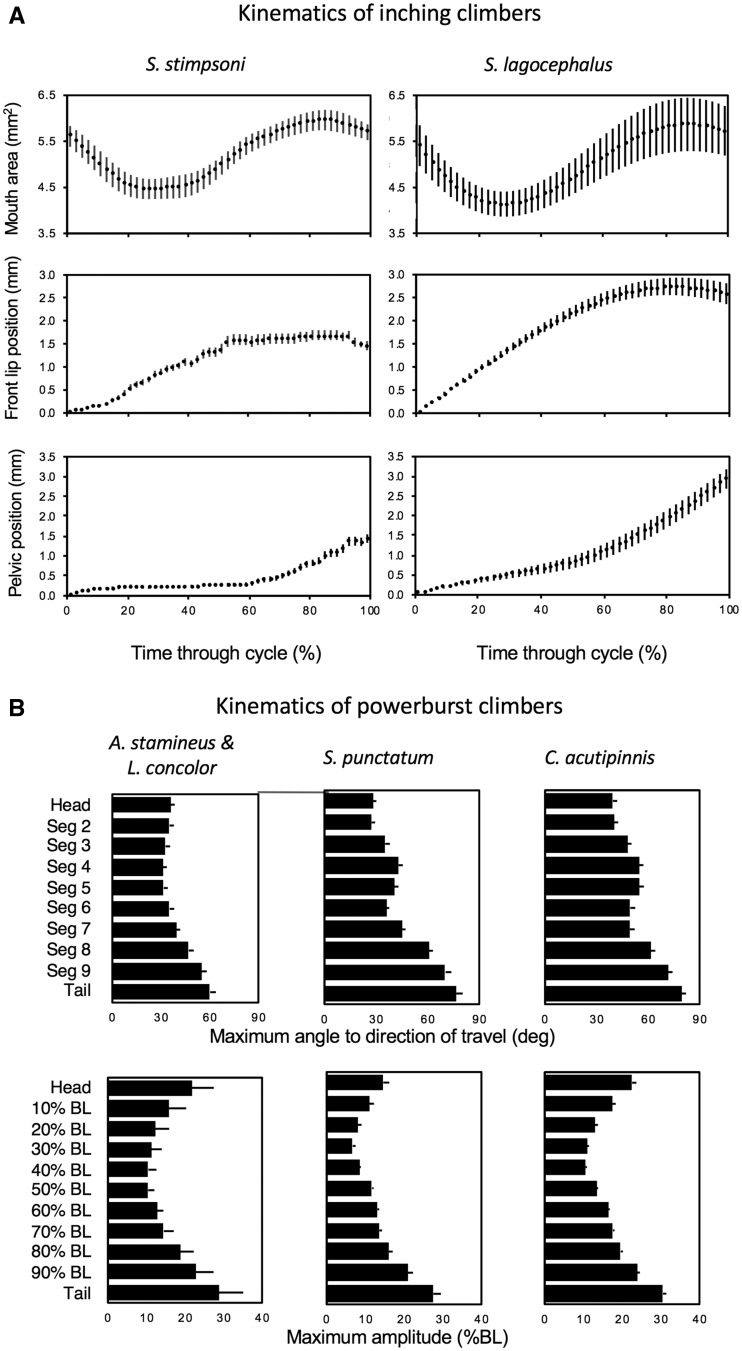
Comparative kinematics of juvenile waterfall-climbing gobies. (**A**) Inching climbers. Mean profiles for kinematic variables during inching climbing by *S. stimpsoni* from Hawai’i (*n* = 26 climbing cycles, left column; data from Schoenfuss and Blob 2003) and *S. lagocephalus* from La Réunion: (*n* = 86 climbing cycles, right column). All trials were normalized to the same time duration, and plots show mean ± SE for every 2% increment of locomotor cycle duration. (**B**) Powerburst climbers. Mean profiles for axial kinematics during vertical powerburst climbing by juvenile *A. stamineus* and *L. concolor* from Hawai’i (*n* = 17 climbing cycles, left column; data pooled for these species as reported by Schoenfuss and Blob 2003), *S. punctatum* from Dominica (*n* = 22 climbing cycles, middle column; data from Schoenfuss et al. 2011); and *C. acutipinnis* from La Réunion (*n* = 87 climbing cycles, right column). Top row: bars for each equal-length segment of the body plot the mean (± SE) maximum angle of that segment to the direction of travel at any point during the cycle. Bottom row: mean (± SE) maximum amplitudes throughout the climbing cycle for each of 11 equally spaced points along the length of the fish, normalized as a percentage of body length. Original data reported in Supplementary Table S1.

Inching *S. lagocephalus* from La Réunion showed similar kinematic profiles to patterns observed previously in *S. stimpsoni* from Hawai’i (Fig. 3A;Supplementary Movies S1–S2). The area of the oral sucker initially decreases as the front lip advances early in the cycle. However, the area of the oral sucker then increases as fish reattach it to the substrate while the pelvic sucker advances up the surface. Ranges of values for kinematic variables are also similar between the species, with the slightly longer upward advance of the oral and pelvic suckers during each cycle in *S. lagocephalus* corresponding to the slightly larger average body size of this species (25 mm standard length) compared to *S. stimpsoni* (22 mm; Table 1; Schoenfuss and Blob 2003). Kinematic profiles across powerburst climbers also show generally similar patterns, with moderate ranges of variation in kinematic variables (Fig. 3B;Supplementary Movies S3–S4). In Hawaiian powerburst climbers, *Sicydium punctatum* from the Caribbean and *C. acutipinnis* from La Réunion, body segments near the head and tail show the greatest amplitudes during axial undulation, with caudal body segments showing the greatest angles to the direction of travel (Fig. 3B). However, larger *C. acutipinnis* (16 mm) and *S. punctatum* (19 mm) exhibit larger values for both of these variables than the smaller (14 mm) Hawaiian taxa.

**Table 1.** Means (± SD) of morphological and performance variables for juveniles of waterfall climbing stream gobies from Hawai’i (*A. stamineus*, *L. concolor*, *S. stimpsoni*), Dominica (*S. punctatum*) and La Réunion (*C. acutipinnis*, *S. lagocephalus*), with numbers in parentheses indicating the sample size of locomotor events from which data were collected with each cycle from a different individual

	Hawai’i			Dominica	La Réunion		Kruskal–Wallis *P*-value
Variable	*Awaous stamineus*	*Lentipes concolor*	*Sicyopterus stimpsoni*	*Sicydium punctatum*	*Cotylopus acutipinnis*	*Sicyopterus lagocephalus*	
Fish length (cm)^1^	1.4 (12)	1.4 (17)	2.2 (17)	1.9 (39)	1.6±0.5 (11)	2.5±0.4 (62)	n/a
Climbing bout duration (s)^1^^,2^	1.81±0.85^a^(12)	1.97±0.7^a^(17)	7.55±6.5^b^(17)	2.51±1.85^a^(39)	2.49±0.52^ac^(11)	3.34±1.51^c^(62)	<0.001
Net climbing speed including rest (cm/s)^1^^,2^	0.21±0.10^abc^(12)	0.21±0.09^b^(17)	0.22±0.07^b^(17)	0.21±0.13^ab^(39)	0.09±0.03^c^(11)	0.28±0.11^ab^(62)	<0.001
Net climbing speed including rest (BL/s)^1^^,2^	0.15±0.07^a^(12)	0.15±0.06^a^(17)	0.10±0.03^ab^(17)	0.11±0.07^ab^(39)	0.06±0.03^b^(11)	0.11±0.05^a^(62)	<0.001
Speed during climbing only (cm/s)^1^^,2^	1.17±0.46^ab^(12)	0.94±0.14^a^(17)	0.34±0.07^bc^(17)	1.02±0.47^a^(39)	0.73±0.25^a^(11)	0.62±0.12^c^(62)	<0.001
Speed during climbing only (BL/s)^1^^,2^	0.84±0.33^a^(12)	0.67±0.10^a^(17)	0.15±0.03^b^(17)	0.54±0.25^a^(39)	0.50±0.23^a^(11)	0.25±0.07^c^(62)	<0.001
Percent time in motion^1–3^	20.0±10.3^a^(12)	22.3±11^a^(17)	54.3±21^b^(16)	21.6±11.6^a^(39)	13.5±5.4^a^(11)	47.2±18.4^b^(62)	<0.001

1 Data for Hawaiian species from (Blob et al. 2006), data for *S. punctatum* from (Schoenfuss et al. 2011), data for species from La Réunion new in this study.

2 Superscript letters (a, b, c) indicate groupings of significantly different species (*P* < 0.05), based on Dunn’s post-hoc tests corrected for multiple comparisons, conducted after Kruskal–Wallis analyses.

3 Data arcsine transformed prior to Kruskal–Wallis analysis.

n/a, not applicable; BL/s, body lengths per second.

**Fig. 4:**
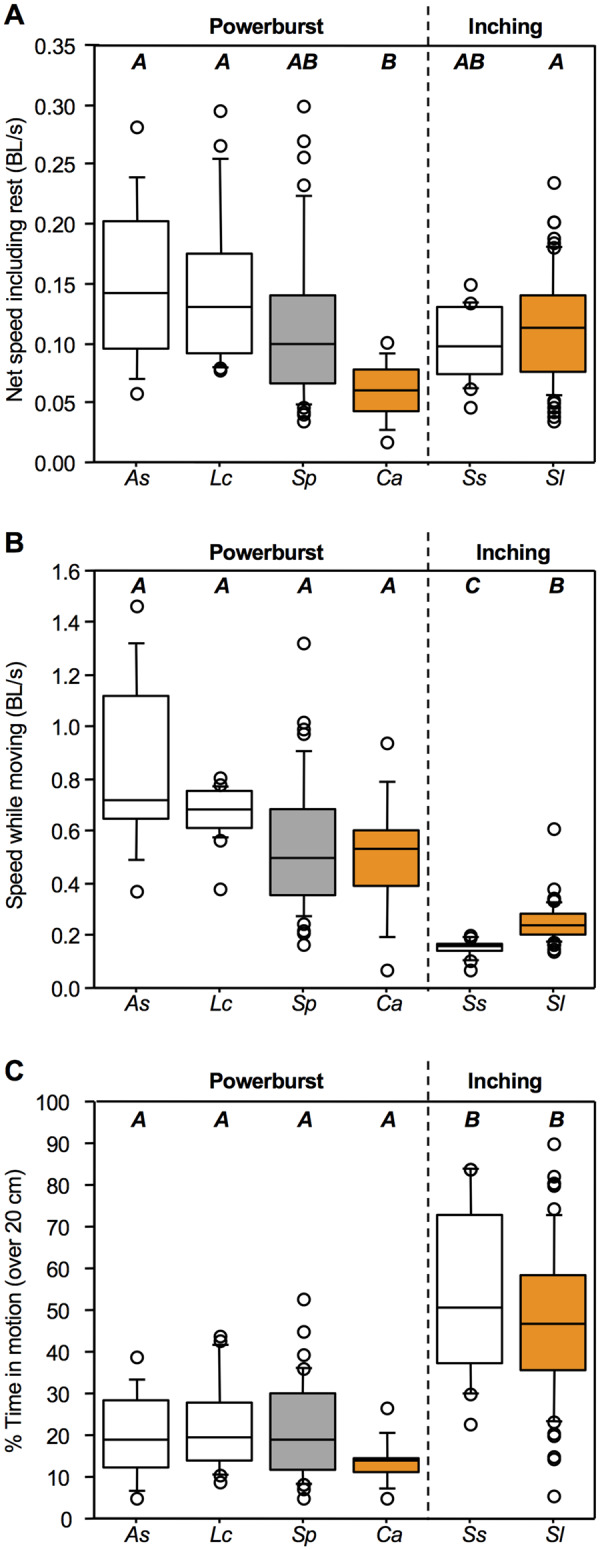
Comparative climbing performance of juvenile waterfall-climbing gobies, measured over 20 cm distances. (**A**) Net speed including periods of rest, normalized for body size. (**B**) Speed during periods of movement only, normalized for body size. (**C**) Proportion of time spent moving during 20 cm trials. Boxes show 25th percentile, median, and 75th values; whiskers illustrate 10th and 90th percentile values; open circles indicate values outside these percentiles. Vertical dashed line in each plot divides species by climbing style (powerburst climbers on the left, inching climbers on the right). Colors represent differences in locality, with white boxes showing data from Hawaiian species derived from Blob et al. (2006), gray boxes data from Caribbean species derived from Schoenfuss et al. (2011), and orange boxes new data from Réunionese species. Different boldface letters above each box plot indicate significant differences between groups, determined by Kruskal–Wallis analyses with Dunn’s post-hoc tests, corrected for multiple comparisons (*P *<* *0.05). *As*, *A. stamineus*; *Lc*, *L. concolor*; *Sp*, *S. punctatum*; *Ca*, *C. acutipinnis*; *Ss*, *S. stimpsoni*; *Sl*, *S. lagocephalus*. Original data and sample sizes are reported in Supplementary Table S2.

In contrast to kinematic comparisons, the two climbing styles show different patterns of variation in performance across taxa. With regard to net climbing speed over 20 cm, including periods of motion and rest, the two species of inching climbers show similar performance to each other (and to three of the four powerburst climbers: mean 0.10–0.14 BL s^−1^; Table 1; Fig. 4A). In contrast, the powerburst climber *C. acutipinnis* from La Réunion (mean 0.06 BL s^−1^) is significantly slower than two of the other powerburst climbers (*Awaous stamineus* and *Lentipes concolor*), as well as one of the inching species (*S. lagocephalus*) (Dunn’s post-hoc test *P* < 0.05; Table 1; Fig. 4A). Thus, powerburst climbers include species with at least two distinct levels of net climbing performance, whereas inching climbers include only one such group. Across all of the variables that we compared, the average coefficient of variation was also greater for powerburst climbing species (45 ± 3.1 standard error [SE]) than for inching species (33 ± 3.2 SE) (Mann–Whitney U-test, *P* < 0.01). However, this apparent greater performance variation among powerburst climbers is a product of the variables that underlie this performance. Comparisons of movement speed (i.e., speed restricted to periods of actual movement) show significant differences between the two inching climbers, whereas the powerburst climbers group similarly (Table 1; Fig. 4B). It is noteworthy, though, that *C. acutipinnis* uses the slowest movement speeds among powerburst species (Table 1; Fig. 4B). Comparisons of the time spent in motion over 20 cm show distinctions between the powerburst (<25%) versus inching (∼50%) climbers (*P* < 0.05), with species within each climbing style grouping together (Table 1; Fig. 4C). However, both *C. acutipinnis* and *S. lagocephalus* from La Réunion showed lower mean values than other species using their respective climbing styles. As a result, the similar net speeds of inching climbers seem to arise through different pathways: *S. stimpsoni* from Hawai’i move more slowly than *S. lagocephalus* from La Réunion, but may spend more time moving. In contrast, the cumulative effect of seemingly minor differences in both movement speed and the portion of time spent moving, differences that were not initially recognized as significant, led to distinctly slow net speeds for *C. acutipinnis* among powerburst species.

## Discussion

Recently evolved novelties have had less time to accrue evolutionary changes that might contribute to functional diversity than older traits. As a result, older traits might be expected to show more variation than recent evolutionary novelties (Wainwright and Price 2016). However, our results from climbing gobies illustrate how the equivalent performance of alternative functional pathways can influence interpretations of the relative diversity exhibited by functional traits. Comparisons of net climbing speed between inching and powerburst species gave an initial indication that more recently evolved climbing by inching was largely similar in performance across species, whereas the older trait of powerburst climbing displayed greater variation across the taxa that retain this climbing mode (Table 1; Fig. 4A). In contrast, comparisons of the mechanisms that underlie the performance of both styles present a different picture, in which powerburst climbing species might show more consistent average patterns than inchers (Fig. 4B). These patterns result because, within each climbing style, species appear to use largely similar kinematics in different ways—for example, by spending more (or less) time moving compared to resting (Fig. 4C). Thus, without considering the mechanics that underlie performance, the functional diversity present within more recently evolved inching might have been masked. Such masking could lead to a potentially oversimplified conclusion that functional diversity is lower in the more recent evolutionary novelty.

The complexity of many functional systems opens opportunities for similar performance to be produced through multiple pathways. One variety of this general phenomenon that has received considerable attention is the many-to-one mapping of structure to function, in which multiple anatomical arrangements of structures may produce equivalent functional performance (Alfaro et al. 2005; Wainwright et al. 2005). Our data from waterfall-climbing gobies provide an example of the broader phenomenon of which many-to-one mapping of structure to function is a part, in which alternative functional pathways—such as different climbing styles, or different patterns of motion versus rest within a climbing style—can produce equivalent locomotor performance. For example, three of the four powerburst species compared show net climbing speeds that can be statistically grouped with those of both inching species observed, despite using different propulsive mechanisms to scale waterfalls (Table 1; Fig. 4A). In addition, among inching species, equivalent climbing performance can be achieved by longer durations of slow movement (e.g., *S. stimpsoni*) and shorter durations of faster movement (e.g., *S. lagocephalus*; Table 1; Fig. 4).

The ultimate similarity of net climbing performance between the two broad styles of climbing in gobies is striking. Out of the six species we compared, from islands in three different ocean basins (Pacific, Caribbean, and Indian), five showed net climbing speeds between 0.10 and 0.14 BL s^−1^. Only *C. acutipinnis* used markedly slower speeds (0.06 BL s^−1^), which were a product of moderately slower movement and a smaller proportion of time spent moving, but which still overlapped with the net speeds of some of the other five species (Fig. 4). Given that climbing behaviors are components of a migratory phase of the life cycle, to what extent might differences in performance impact features such as the in-stream distributions and upstream penetration of species? In La Réunion, *C. acutipinnis* has a higher failure rate during attempted climbs than faster *S. lagocephalus* (Lagarde et al. 2018), and it takes *C. acutipinnis* 20–25% more days to migrate to adult upstream habitats (Lagarde et al. 2020). Although this could allow faster juvenile *S. lagocephalus* to reach suitable habitat before *C. acutipinnis*, both species ultimately penetrate to comparable upstream distances (Teichert et al. 2014). Hawaiian species of amphidromous gobies show even more limited correlations between climbing performance and in-stream distribution. Although the two Hawaiian powerburst species exhibit nearly identical net speeds, one species (*A. stamineus*) penetrates the shortest distance upstream and the other species (*L. concolor*) penetrates the furthest, with inching *S. stimpsoni* reaching intermediate distances between the two (Schoenfuss and Blob 2003, 2007). Given that climbing performance does not predict ecological factors such as habitat distribution, changes in net performance appear unlikely to have formed a selective basis for the evolutionary origin of inching as a functional novelty in climbing style. Instead, the origin of inching with an oral sucker may have been tied to non-locomotor pressures, potentially reflecting an exaptation of feeding adaptations for locomotor roles (Cullen et al. 2013).

## Authors’ contributions

R.W.B., R.L., and H.L.S. designed the study and collected data. All authors contributed to data analysis, interpretation, and manuscript development, and approved the final manuscript.

## Supplementary Material

obz029_Supplementary_Data
